# The impact of psychosocial safety climate on the intervention effect of psychotherapeutic consultation at work in Germany – secondary analysis of a randomized controlled trial

**DOI:** 10.1186/s12889-025-24394-5

**Published:** 2025-10-22

**Authors:** Meike Heming, Nicole Hander, Harald Gündel, Manuel Feißt, Marieke Hansmann, Christoph Kröger, Nadine Mulfinger, Jeannette Weber, Regina Herold, Ute Schröder, Uta Wegewitz, Peter Angerer

**Affiliations:** 1 Institute of Occupational, Social, and Environmental Medicine, Centre for Health and Society, Medical Faculty and University Hospital Düsseldorf, Heinrich-Heine University Düsseldorf, Moorenstraße 5, Düsseldorf, 40225 Germany; 2 Department of Psychosomatic Medicine and Psychotherapy, Ulm University Medical Center, Albert-Einstein-Allee 23, Ulm, Baden-Württemberg, 89071 Deutschland; 3 Institute of Medical Biometry, Heidelberg University, Heidelberg, Germany; 4 Institute of Psychology, University of Hildesheim, Universitätsplatz 1, Hildesheim, 31141 Germany; 5 Department Psychiatry II, Section of Health Economics and Psychiatric Services Research, Ulm University & BKH Günzburg, Lindenallee 2, Günzburg, 89312 Germany; 6 Department of Psychosomatic Medicine and Psychotherapy, University Hospital of Erlangen, Friedrich-Alexander University Erlangen-Nürnberg (FAU), Erlangen, 91054 Germany; 7 Federal Institute for Occupational Safety and Health (BAuA) Division 3 Work and Health Unit 3.5 Evidence-based Occupational Health, Workplace Health Management, Berlin, Germany; 8 Federal Institute for Occupational Safety and Health (BAuA) Division 3 Work and Health, Workplace Health Management, Berlin, Germany

**Keywords:** Psychosocial safety climate, Psychotherapy, Workplace, Mental health, Work stress, Occupational health, Psychotherapeutic consultation at work

## Abstract

**Background:**

Psychotherapeutic consultation at work (PT-A) aims to reach employees at risk of or suffering from common mental disorders early by offering low-threshold consultation that combines person-related and work-related modules. In addition to this individual-level approach, it is suggested that the psychosocial safety climate (PSC) in an organization precedes psychosocial working conditions and impacts employees' psychological health. The PSC reflects employees' perceptions regarding the extent to which psychological health and safety are prioritized by the management. However, little is known about the role of PSC on the intervention effect in participants receiving PT-A. Therefore, this study aims to investigate whether PSC moderates the effect of PT-A on self-reported depressive symptoms, anxiety, and general health status.

**Methods:**

As a secondary analysis, this study analyzed data on participants (*n* = 549) from a multicenter randomized controlled trial in Germany evaluating the effectiveness of PT-A. Participants completed a baseline questionnaire (T0) and follow-up questionnaires nine months (T1) and 15 months later (T2). They provided information on general health status (one item of the 12-items Veterans RAND health survey), depressive symptoms (Patient-Health-Questionnaire-9), anxiety symptoms (Generalized Anxiety Disorder Scale-2) and the four-item PSC questionnaire. To investigate the role of PSC for the intervention effect, an interaction term (PSC*treatment group) was included in multiple linear regression analyses estimating (mental) health indicators at either T1 (*n* = 411) or T2 (*n* = 362). Analyses were adjusted for sex, age and occupational level.

**Results:**

Participants in the intervention group reported a significant symptom reduction nine and 15 months after PT-A. In terms of direct effects, PSC was negatively associated with anxiety symptoms at T1 (B = 0.16, *p* = .04). PSC did not moderate the intervention effect on depressive symptoms, anxiety symptoms or general health status.

**Conclusions:**

Findings indicated a significant symptom reduction after PT-A, and showed that PSC does not play a role in the extent of symptom reduction among employees in Germany. However, PSC may need to be improved in German workplaces and its effect on individual-level symptoms regarding common mental disorders (CMD) should be further investigated.

**Trial registration:**

The RCT was registered at the German Clinical Trial Register on 01.03.2021 (DRKS00023049) https//drks.de/search/en/trial/DRKS00023049.

**Supplementary Information:**

The online version contains supplementary material available at 10.1186/s12889-025-24394-5.

## Background

Poor psychosocial working conditions refer to a combination of social and psychological factors related to work or the work environment and are known to be associated with common mental disorders (CMD) such as depression [1]. However, psychosocial working conditions in the workplace are largely shaped and determined by the management and leaders of companies. It is therefore important to consider not only the conditions but also management’s efforts to promote health at work and their impact from the perspective of the employees. This so-called Psychosocial Safety Climate (PSC) is defined as “policies, practices, and procedures for the protection of worker psychological health and safety” [2, p.580]]. It reflects an organizational climate that focuses on psychological health, but also takes into account physical health and safety at work [2]. The PSC contains four domains that are primarily determined by values and actions of the management in organizations towards improvement of psychological health of the employees [2–4]. PSC in this study is defined here as employees’ subjective beliefs or representations of these policies, practices and procedures.

According to the PSC framework, PSC precedes and influences working conditions and, if low, directly contributes to poorer mental health [2]. Research has shown, that components of the PSC, such as corporate communication about mental health or participation of employees in work stress prevention, are negatively correlated with depressive symptoms, especially in larger companies [5]. Research has also shown that the association between unit level PSC and psychological strain was mediated by working conditions such as control or emotional demands in nurses [6]. Other longitudinal research has shown that a high PSC can reduce the risk of experiencing bullying and ultimately psychological health problems at work through implemented procedures such as stress reduction or conflict resolutions (i.e. enacted PSC) [3]. Furthermore, organizational PSC moderated the positive association between bullying and psychological health problems among Australian workers showing that within high PSC the association was attenuated [7]. A diary study among schoolteachers showed that PSC moderated a negative relationship between daily recovery of work stress and fatigue while also showing a main effect on fatigue [8]. These findings suggest that a high PSC may act as a buffer to protect employees’ mental health [7, 8].

Whereas PSC is a concept related to mental health in a company, employees’ mental health in the companies also depends on the health care system in the country where the company is located. In Germany there is a mismatch between treatment needs and utilization, and long waiting times for psychotherapy are observed [9–11]. The most common diagnoses for disability pensions in Germany belong to the group of CMD [12], and poor working conditions are known to increase the risk of developing depressive symptoms over time [13]. This highlights the need for additional services for employees that can help individuals who suffer from, or are at risk of developing, CMD and thus are at risk of impaired work ability/(long-term) sickness absence. The multicenter randomized-controlled trial (RCT) called “friaa” (early intervention in the workplace, in German: Frühe Intervention am Arbeitsplatz) tried to fill this treatment gap by offering low-threshold psychotherapeutic consultation at work (PT-A) without long waiting times to interested employees [14]. PT-A offers additional support to regular healthcare by providing first consultation, diagnosis, treatment and, if needed, therapeutic support upon returning to work. It is provided by psychotherapists who focus on private- as well as work-related topics within the sessions [14]. Previous evidence from similar interventions has shown that employees can be reached at an early stage of symptom reporting [15] and that depressive, anxiety and somatic symptoms decrease [16]. Individuals with work-related mental health problems showed reductions of depressive and anxiety symptoms and emotional exhaustion in group therapy focused on work-related aspects [17]. The friaa study showed that the PT-A did not significantly reduce self-reported sickness absence days in the intervention group but showed effectiveness of PT-A in terms of self-reported psychological constructs such as depressive and somatic symptoms, anxiety symptoms, and return to work self-efficacy (Rothermund-Nassir et al.: Effectiveness of psychotherapeutic consultation at work compared to care as usual for employees with common mental disorders or subthreshold symptoms: A randomized controlled multicenter trial in Germany, accepted/forthcoming).

Following the PSC framework [2], the organizational climate may also influence the extent of a potential success achieved by PT-A addressing individual factors and psychosocial working conditions. As PSC is supposed to protect employees’ psychological health, it seems reasonable, that a certain level of PSC at work may modify the PT-A’s effectiveness [18]. For example, in a context where employees perceive a high PSC, they may report more symptom reduction or improved general health status after participating in PT-A, as they might feel better supported or equipped by their management to transfer their gained strategies or individual solutions into the workplace. Thus, a high PSC may entail good leadership styles on all levels of a company, which can promote psychological health of employees [19]. Feeling equipped and having sufficient resources to fulfill workplace demands is important for employee well-being and motivation and can be achieved within a good PSC [2]. Thus, a direct effect of PSC on employee’s health may also impact the magnitude of an intervention effect after participation in PT-A. A similar line of thought was applied in a study that investigated whether PSC moderated the effectiveness of an Employee Assistance Program (EAP) in Australia and New Zealand among 25 employees [18]. EAPs were originally developed for companies to advice employees on drug and alcohol abuse [20]. Today, the range of services can entail problem evaluation, brief advice including referral to other instances for legal or financial advice, and also support for family members of employees [20, 21]. In contrast to PT-A, EAP is provided by several professions, such as social workers or counsellors [22]. Results showed that PSC moderated the association between mental health or well-being before and after EAP: High PSC contributed to a greater improvement of mental health and well-being after EAP, compared to a low PSC [18]. The authors could also show a main effect of PSC on mental health and well-being, indicating that PSC is directly associated with employees’ health status [18].

Whether the effect of PT-A on (mental) health outcomes is moderated by PSC has not been investigated so far. Potential success of PT-A may be restricted, if the corporate climate is not supporting or enabling structures to improve employees’ psychological health [18]. There may be a lack of motivation/willingness to implement individual solutions under a poor PSC, which would weaken the intervention effect. Therefore, we would like to investigate whether PSC acts as a moderator for the effect of PT-A on depressive symptoms, anxiety symptoms or general health status in a sample of employees in Germany. Expanding research on this topic may help to tailor interventions addressing mental health at the workplace and to boost its impact, respectively.

## Methods

### Study design and study sample

Data from participants (*n* = 549) in the RCT called ‘friaa’ were investigated in this study and used for this exploratory approach [14]. Companies were recruited from five universities located in Germany according to a proximity principle. Employees started the intervention between September 2021 and January 2023. Employees were mainly recruited via their companies: the study was advertised through various strategies, such as flyers, intranet and mailing lists, and by various company stakeholders, such as occupational health services, supervisors or social counselling. Companies of different sizes were included in the study if they had no other comparable programs for their employees during the study period. During the recruitment process, three universities additionally recruited participants via social media. Inclusion criteria for participation were a minimum age of 18 years and being employed for at least 15 h per week. Reporting of subclinical symptoms of CMD or a diagnosis of CMD was another inclusion criterion, which was assessed by the psychotherapists at the first diagnostic session. Exclusion criteria included diagnoses of substance abuse, psychosis, schizophrenia, other severe somatic health conditions, current psychotherapeutic treatment and application for retirement pension. For a detailed description of study design and study population, see Weber et al. [14]. All participating universities received ethical approval from their ethics committees: Ulm University (November 2020, 339/20), Friedrich-Alexander University Erlangen-Nürnberg (December 2020, 525_20 Bc), University of Hildesheim (January 2021, 165) and Heinrich-Heine-University Düsseldorf (January 2021, 2021–1279) [14]. All participants provided written informed consent.

All participants received one diagnostic session encompassing 100 min with a psychotherapist. Afterwards, randomization by 1:1 allocation conducted by an external institute divided the participants into either intervention or control group. The control group received recommendations for regular healthcare and a supportive telephone call from the psychotherapist three months after the initial appointment. Participants in the intervention group could further receive up to 16 sessions of psychotherapeutic consultation within a period of nine months: another eleven treatment sessions and five sessions of therapeutic support for employees that returned to work after being on sick leave for several weeks due to the mental problem. The sessions covered both private and work-related topics within an adapted modular based approach by Bode et al. [23]. All participants filled in a baseline questionnaire before receiving PT-A, covering several health-related, work-related and socioeconomic questions (T0). Participants were asked to fill in follow-up questionnaires nine months after T0 (T1) and 15 months after T0 (T2). Most of the questionnaires were answered online, in individual cases universities offered paper-pencil procedures. The online versions included some mandatory questionnaires.

The baseline questionnaire (T0) was filled in by 549 participants. As only one person reported a diverse gender and one person had missing values for every question, these two participants could not be included in the analyses (*n* = 547). The first follow-up questionnaire (T1) was filled in by 420 participants and thus were eligible for analyses considering T1 (response rate = 76.5%). The second follow-up questionnaire (T2) was filled in by 372 participants (response rate = 67.8%). In the analyses only participants were considered who had no missing values in the study variables, which resulted in a final study sample of *n* = 411 for T1 analyses and *n* = 362 for T2 analyses.

### Measures

The general health status was assessed using one item of the German version of the Veterans RAND 12-Item Health Survey (VR-12) questionnaire at T0, T1 and T2 [24–26]. This item asks the respondents to describe their state of health in general, using a five-point Likert scale ranging from one (excellent) to five (poor). The scores are recoded into values between zero and 100 where one is recoded to 100, reflecting excellent health [26]. Thus, higher scores reflect a better general health status.

Depressive symptoms were assessed at T0, T1 and T2 with the German version of the Patient-Health-Questionnaire (PHQ-9) [27]. Nine items ask about the frequency of criteria for a depressive disorder in the past two weeks. Answers are given on a four-point Likert scale from zero (not at all) to three (nearly every day). A computed sum-score with higher values reflecting more depressive symptoms was used within this study. A sum-score of ten or higher indicates a major depressive disorder, where ten to 14 reflects a moderate depression, 15 to 19 a moderately severe and 20 to 27 a severe depression [27]. Cronbach’s alphas were 0.80 at T0, 0.87 at T1 and 0.86 at T2 indicating good reliability.

Anxiety symptoms were assessed at T0, T1 and T2 with the German version of the Generalized Anxiety Disorder Scale-2 (GAD-2) [28]. Two items are answered on a four-point Likert scale from zero (not at all) to three (nearly every day). A sum-score was computed with higher values reflects more anxiety symptoms. Values greater than or equal to three can indicate clinically relevant anxiety symptoms [28]. Cronbach’s alphas were 0.75 at T0, 0.81 at T1 and 0.82 at T2 indicating acceptable and good reliability.

The PSC was assessed at T0 with the shortened four item version [2]. The items reflect the four dimensions of the 12-item version [29] with one question for each dimension. The first aspect of PSC assesses the commitment and support that is given by the management towards prevention of stress at work and promotion of mental health [3, 29]. The second aspect of PSC reflects the importance given by the management towards mental health compared to productivity [3, 29]. The third aspect assesses the corporate communication about mental health and work stress and reflects whether and how employees are informed and addressed by the management [3]. The fourth aspect of PSC assesses participation and involvement in work stress prevention [3]. An example item is ‘all layers of the organization are involved in the prevention of stress’ [2]. Answers were given on a five-point Likert scale ranging from zero (does not apply at all) to four (does fully apply). A mean scale was calculated to reflect the overall PSC, with higher scores reflecting higher PSC. The mean scale was calculated when the participants answered at least two items. Cronbach’s alpha was 0.84 at T0 and indicated good reliability. In addition, the sum scale was calculated in order to show the distribution of existing PSC benchmarks for the study samples [30]. For this purpose only, PSC was aggregated at the company level where data was available (i.e., this was the case for *n* = 340 participants belonging to *n* = 44 companies). Scores > 12 indicate a low risk, scores > 8 to 12 indicate a moderate risk and scores ≤ 8 indicate a high risk to occupational health and safety at work [30].

Several potential confounder variables were considered in the analyses: sex (one refers to male; two refers to female); age (continuously measured); occupational position (indications of occupational positions were dichotomized into blue-collar (zero)/white-collar workers (one)) Blue-collar workers reflect skilled or unskilled blue-collar workers and foremen/master craftsmen. All other workers reflect white-collar workers [31].

### Statistical analyses

Differences on demographic study variables between non-responders at T1 or T2 and responders at T0 were investigated by either chi-square test for binary variables or t-tests for independent samples for continuous variables. Non-responder at T1 (T2) are defined as participants who have filled in the baseline questionnaire but have not started to answer questionnaires at T1 (T2). Correlation analyses were conducted for all study variables.

In multiple linear regression analyses, it was investigated whether PSC moderates the intervention effect on depressive symptoms, anxiety symptoms or general health status for two different time points. Depressive symptoms at either T1 or T2 were used as dependent variable. Depressive symptoms at T0 were included as an independent variable, as well as the treatment group (intervention group/control group) and confounding variables (sex, age and occupational level) in a first model. Further, the PSC variable was included in a second model. In a third model, the interaction term PSC × treatment group was included. Model comparisons were performed using ANOVA to assess whether the inclusion of the interaction term increased the explained variance in the models. These analyses were repeated accordingly for the outcome variable representing anxiety symptoms and general health status. Due to the use of interaction terms, all continuous independent variables were z-standardized. Sensitivity analyses without introducing confounder variables can be seen in the supplemental material (Table S.1). Analyses were conducted with IBM SPSS Statistics version 29. The level of significance was set at *p* =.05, and unstandardized regression coefficients are shown as well as 95% confidence intervals (CI). In addition, power analysis was performed with the R package pwr (function pwr.f2.test). It indicated that the sample size of *n* = 411 (*n* = 362) was sufficient to detect an effect size of f^2^ = 0.019 (f^2^ = 0.022) with a power of 0.80 at significance level of 0.05.

## Results

Table 1 shows the descriptive results of study variables for participants in the intervention and control group with complete data at T1 (*n* = 411) and Table 2 shows the descriptive results of study variables for participants in the intervention and control group with complete data at T2 (*n* = 362). The mean age was approximately 46 years in both groups. The majority of the sample belonged to the occupational group of white-collar workers. At baseline, the study participants in both groups were considered as being moderately depressed and as showing increased anxiety symptoms, on average. At T1 and T2, participants reported mean values of anxiety symptoms below a cut-off for clinically relevant anxiety symptoms and mild depressive symptoms. The mean PSC varied between study samples and groups from 1.57 to 1.62.

**Table 1:** Descriptive results of the study variables for intervention and control group participants at T1 (*n* = 411)

	IG (*n* = 227)			CG (*n* = 184)		
	n (%) or min-max	Mean/ Median	SD	n (%) or min-max	Mean/ Median	SD
Sex						
Female	132 (58.1)			96 (52.2)		
Male	95 (41.9)			88 (47.8)		
Age (y)	21–63	46.27	10.27	22–64	46.35	11.35
Occupational position						
Blue-collar workers	44 (19.4)			42 (22.8)		
White-collar workers	183 (80.6)			142 (77.2)		
PSC	0–4	1.59/ 1.5	0.92	0–4	1.58/ 1.75	0.94
Depressive symptoms T0	2–26	13.12	5.10	2–25	12.41	5.22
Depressive symptoms T1	0–25	7.89	5.39	0–27	9.95	5.70
Anxiety symptoms T0	0–6	3.50	1.71	0–6	3.46	1.76
Anxiety symptoms T1	0–6	2.21	1.67	0–6	2.58	1.73
General health status T0	0–85	47.03	19.66	0–85	49.32	19.14
General health status T1	0-100	54.36	22.15	0-100	52.50	21.45

*PSC* Psychosocial safety climate, *SD* Standard deviation, *IG* intervention group, *CG* control group

**Table 2:** Descriptive results of the study variables for intervention and control group participants at T2 (*n* = 362)

	IG (*n* = 205)			CG (*n* = 157)		
	n (%) or min-max	Mean/ Median	SD	n (%) or min-max	Mean/ Median	SD
Sex						
Female	116 (56.6)			83 (52.9)		
Male	89 (43.4)			74 (47.1)		
Age	21–62	46.48	10.21	22–64	46.59	11.33
Occupational position						
Blue-collar workers	39 (19.0)			31 (19.7)		
White-collar workers	166 (81.0)			126 (80.3)		
PSC	0–4	1.57/ 1.5	0.92	0–4	1.62/ 1.75	0.96
Depressive symptoms T0	2–26	12.83	5.20	2–25	12.26	5.32
Depressive symptoms T2	0–27	7.39	5.14	0–21	8.49	4.77
Anxiety symptoms T0	0–6	3.43	1.73	0–6	3.48	1.81
Anxiety symptoms T2	0–6	1.92	1.56	0–6	2.33	1.65
General health status T0	0–85	47.46	18.49	0–85	49.11	18.21
General health status T2	0-100	58.10	22.24	0-100	55.10	21.55

*PSC* Psychosocial safety climate, *SD* Standard deviation, *IG* intervention group, *CG* control group

Table 3 shows the distribution of the PSC benchmarks (according to [30] in the two study samples at hand, aggregated to the workplace level. About 85% of participants in both groups showed a moderate risk for poor occupational health and safety at their workplace. Around 7% of the participants showed a high risk and about 7% show a low risk, i.e., good occupational health and safety at their workplace.

**Table 3:** Distribution of PSC benchmarks standards and recommendations for available data of study samples at T1 and T2, aggregated on workplace level

PSC benchmarks according to (30)	T1 (*n* = 254)	T2 (*n* = 223)
	%	%
Low risk	7.1	7.2
Moderate risk	85.8	85.2
High risk	7.1	7.6

Correlation analyses for all study variables and all time points are provided in the Supplementary Material (Table S.2). Table S.3 in the Supplementary Material shows the results for the differences between non-responders at T1 or T2 and the responders. There were almost no significant differences between responders and non-responders. Results showed a significant correlation between occupational position and responder groups, χ² [1] = 4.39, *p* =.036, φ = − 0.09. There were more participants in the blue-collar group as expected for non-responders at T2 (*n* = 75 for responder and *n* = 50 for non-responder), and fewer participants in the white-collar group as expected (*n* = 296 for responder and *n* = 127 for non-responder). There was no significant difference of age between responder groups t(320.65) = 1.90, *p* =.058. For example, responder showed a mean age of 46.56 (SD = 10.68) and non-responder at T2 showed a mean age of 44.58 (SD = 11.71).

The results of the regression analyses for depressive symptoms at T1 and T2 are shown in Table 4. Participants in the intervention group reported significantly fewer depressive symptoms at T1 compared with the control group. This remained after adjustment for confounding variables. No main effect was observed for PSC in model 2. The interaction term PSC*group (intervention or control group) introduced in model 3 did not reach the significance level. Similar results were obtained for the study sample of T2. Depressive symptoms at T2 reported by the intervention group were significantly reduced, compared to the control group, also after adjusting for age, sex and job type. In model 2, the inclusion of PSC in the model showed no significant direct effects on depressive symptoms at T1 or T2. Again, the interaction term PSC*group did not reach the significance level.

**Table 4:** Regression analyses predicting depressive symptoms at T1 (*n* = 411) and T2 (*n* = 362)

	Model 1			Model 2			Model 3		
	**Depressive symptoms at T1**								
	B	p	95% CI	B	p	95% CI	B	p	95% CI
Depressive symptoms T0	2.75	0.00	2.29; 3.21	2.72	0.00	2.26; 3.18	2.72	0.00	2.26; 3.18
Group	−2.46	0.00	−3.39; −1.53	−2.46	0.00	−3.39; −1.53	−2.46	0.00	−3.39: −1.54
Age	0.79	0.00	0.31; 1.27	0.76	0.00	0.28; 1.24	0.78	0.00	0.30; 1.26
Sex	0.60	0.21	−0.34; 1.54	0.61	0.20	−0.32; 1.55	0.65	0.18	−0.29; 1.59
occupational position	−0.18	0.76	−1.33; 0.97	−0.20	0.74	−1.35; 0.95	−0.23	0.69	−1.38; 0.92
PSC				−0.35	0.15	−0.82; 0.12	0.58	0.43	−0.88; 2.04
PSC*group							−0.64	0.19	−1.58; 0.31
	Model fit			Model fit			Model fit		
	Adj. R^2^ = 0.285, F = 33.709(5,405), *p* <.001			Adj. R^2^ = 0.287, F = 28.520(6,404), *p* <.001			Adj. R^2^ = 0.288, F = 24.743(7,403), *p* <.001		
				Model comparison 1 with 2			Model comparison 2 with 3		
				R^2^	∆R^2^	F statistics	R^2^	∆R^2^	F statistics
				0.298	0.004	2.112 (1, 404)	0.301	0.003	1.761 (1, 403)
						*p* =.147			*p* =.185
	**Depressive symptoms at T2**								
	B	p	95% CI	B	p	95% CI	B	p	95% CI
Depressive symptoms T0	2.06	0.00	1.61; 2.52	2.06	0.00	1.60; 2.51	2.06	0.00	1.60; 2.51
Group	−1.32	0.01	−2.26; −0.38	−1.33	0.01	−2.27; −0.39	−1.34	0.01	−2.28; −0.41
Age	0.90	0.00	0.42; 1.39	0.89	0.00	0.41; 1.38	0.91	0.00	0.42; 1.39
Sex	−0.08	0.87	−1.03; 0.87	−0.07	0.88	−1.02; 0.87	−0.05	0.93	−0.99; 0.90
occupational position	−0.15	0.81	−1.34; 1.04	−0.15	0.80	−1.34; 1.04	−0.21	0.73	−1.40; 0.98
PSC				−0.12	0.62	−0.59; 0.35	0.91	0.22	−0.54; 2.36
PSC*group							−0.71	0.14	−1.65; 0.23
	Model fit			Model fit			Model fit		
	Adj. R^2^ = 0.199, F = 18.972(5,356), *p* <.001			Adj. R^2^ = 0.198, F = 15.818(6,355), *p* <.001			Adj. R^2^ = 0.200, F = 13.971(7,354), *p* <.001		
				Model comparison 1 with 2			Model comparison 2 with 3		
				R^2^	∆R^2^	F statistics	R^2^	∆R^2^	F statistics
				0.211	0.001	0.250 (1, 355)	0.216	0.005	2.190 (1, 354)
						*p* =.618			*p* =.140

Group: Control group as reference. Sex: male as reference. Occupational position: blue-collar workers as reference

*B* unstandardized regression coefficient, *P* p-value, *PSC *psychosocial safety climate *CI* confidence interval

Table 5 shows the results of the regression analyses for anxiety symptoms at T1 and T2. Similar to the results for depressive symptoms, lower levels of anxiety symptoms can be observed in the intervention, compared to the control group at both time points. The introduction of PSC in model 2, shows a significant negative main effect of PSC on anxiety symptoms at T1 but not at T2. The subsequent introduction of the interaction term PSC*group did not result in a significant moderation effect of PSC at either time point.

**Table 5:** Regression analyses predicting anxiety symptoms at T1 (*n* = 411) and T2 (*n* = 362)

	Model 1			Model 2			Model 3		
	**Anxiety symptoms at T1**								
	B	p	95% CI	B	p	95% CI	B	p	95% CI
Anxiety symptoms T0	0.66	0.00	0.51; 0.81	0.66	0.00	0.51; 0.80	0.65	0.00	0.50; 0.80
Group	−0.40	0.01	−0.71; −0.10	−0.40	0.01	−0.70; −0.10	−0.40	0.01	−0.70; −0.10
Age	0.14	0.07	−0.01; 0.30	0.13	0.09	−0.02; 0.29	0.13	0.09	−0.02; 0.29
Sex	0.21	0.18	−0.10; 0.52	0.22	0.16	−0.09; 0.53	0.22	0.16	−0.09; 0.53
occupational position	0.09	0.65	−0.29; 0.46	0.08	0.67	−0.29; 0.45	0.08	0.67	−0.29; 0.45
PSC				−0.16	0.04	−0.31; −0.01	−0.11	0.66	−0.58; 0.37
PSC*group							−0.04	0.82	−0.35; 0.27
	Model fit			Model fit			Model fit		
	Adj. R^2^ = 0.172, F = 18.022(5,405), *p* <.001			Adj. R^2^ = 0.178, F = 15.838(6,404), *p* <.001			Adj. R^2^ = 0.176, F = 13.551(7,403), *p* <.001		
				Model comparison 1 with 2			Model comparison 2 with 3		
				R^2^	∆R^2^	F statistics	R^2^	∆R^2^	F statistics
				0.190	0.008	4.202(1, 404)	0.191	0.000	0.055 (1,403)
						*p* =.041			*p* =.816
	**Anxiety symptoms at T2**								
	B	p	95% CI	B	p	95% CI	B	p	95% CI
Anxiety symptoms T0	0.64	0.00	0.50; 0.79	0.64	0.00	0.49; 0.79	0.64	0.00	0.50; 0.79
Group	−0.40	0.01	−0.71; −0.10	−0.40	0.01	−0.71; −0.10	−0.40	0.01	−0.71; −0.10
Age	0.15	0.07	−0.01; 0.30	0.14	0.07	−0.01; 0.30	0.14	0.08	−0.01; 0.30
Sex	0.14	0.36	−0.16; 0.45	0.14	0.36	−0.16; 0.45	0.14	0.38	−0.17; 0.45
occupational position	0.13	0.51	−0.25; 0.51	0.13	0.51	−0.25; 0.51	0.13	0.49	−0.25; 0.52
PSC				−0.02	0.79	−0.17; 0.13	−0.13	0.58	−0.60; 0.34
PSC*group							0.08	0.62	−0.23; 0.38
	Model fit			Model fit			Model fit		
	Adj. R^2^ = 0.187, F = 17.661(5,356), *p* <.001			Adj. R^2^ = 0.185, F = 14.691(6,355), *p* <.001			Adj. R^2^ = 0.184, F = 12.601(7,354), *p* <.001		
				Model comparison 1 with 2			Model comparison 2 with 3		
				R^2^	∆R^2^	F statistics	R^2^	∆R^2^	F statistics
				0.199	0.000	0.70 (1, 355)	0.199	0.001	0.248 (1, 354)
						*p* =.792			*p* =.619

Group: Control group as reference. Sex: male as reference. Occupational position: blue-collar workers as reference

*B *unstandardized regression coefficient*, P p-*value*, PSC *psychosocial safety climate*, CI *confidence interval

Table 6 shows the results of the regression analyses for general health status at T1 and T2. Participants in the intervention group reported a better general health status at T2 compared to the control group. This intervention effect was not significant at T1. The introduction of PSC in model 2 at both time points shows no significant direct effects of PSC on general health status. The inclusion of the interaction term PSC*group in model 3 also showed no significant moderation effect of PSC at either time point.

**Table 6:** Regression analyses predicting general health status at T1 (*n* = 411) and T2 (*n* = 362)

	Model 1			Model 2			Model 3		
	**General health status at T1**								
	B	p	95% CI	B	p	95% CI	B	p	95% CI
General health status T0	9.50	0.00	7.66; 11.34	9.48	0.00	7.64; 11.32	9.48	0.00	7.63; 11.32
Group	3.05	0.10	−0.59; 6.70	3.05	0.10	−0.60; 6.69	3.05	0.10	−0.61; 6.70
Age	−4.41	0.00	−6.34; −2.49	−4.37	0.00	−6.30; −2.44	−4.37	0.00	−6.31; −2.44
Sex	−4.45	0.02	−8.14; −0.77	−4.48	0.02	−8.17; −0.79	−4.49	0.02	−8.18; −0.79
occupational position	5.18	0.02	0.67; 9.70	5.21	0.02	0.69; 9.73	5.22	0.02	0.69; 9.75
PSC				0.64	0.49	−1.21; 2.50	0.43	0.88	−5.31; 6.16
PSC*group							0.15	0.94	−3.57; 3.87
	Model fit			Model fit			Model fit		
	Adj. R^2^ = 0.273, F = 31.782(5,405), *p* <.001			Adj. R^2^ = 0.272, F = 26.528(6,404), *p* <.001			Adj. R^2^ = 0.270, F = 22.683(7,403), *p* <.001		
				Model comparison 1 with 2			Model comparison 2 with 3		
				R^2^	∆R^2^	F statistics	R^2^	∆R^2^	F statistics
				0.283	0.001	0.468 (1, 404)	0.283	0.000	0.006 (1, 403)
						*p* =.494			*p* =.937
	**General health status at T2**								
	B	p	95% CI	B	p	95% CI	B	p	95% CI
General health status T0	9.21	0.00	7.09; 11.32	9.21	0.00	7.09;11.33	9.20	0.00	7.08; 11.32
Group	3.79	0.06	−0.22; 7.80	3.79	0.06	−0.23;7.81	3.82	0.06	−0.21; 7.84
Age	−5.19	0.00	−7.30; −3.08	−5.19	0.00	−7.31; −3.07	−5.21	0.00	−7.33; −3.09
Sex	−2.05	0.32	−6.11; 2.01	−2.05	0.32	−6.11;2.02	−2.10	0.31	−6.17; 1.97
occupational position	3.64	0.16	−1.45; 8.72	3.64	0.16	−1.45;8.73	3.73	0.15	−1.37; 8.83
PSC				−0.01	0.99	−2.02;2.01	−1.80	0.57	−8.02; 4.43
PSC*group							1.23	0.55	−2.82; 5.28
	Model fit			Model fit			Model fit		
	Adj. R^2^ = 0.235, F = 23.198(5,356), *p* <.001			Adj. R^2^ = 0.233, F = 19.277(6,355), *p* <.001			Adj. R^2^ = 0.232, F = 16.544(7,354), *p* <.001		
				Model comparison 1 with 2			Model comparison 2 with 3		
				R^2^	∆R^2^	F statistics	R^2^	∆R^2^	F statistics
				0.246	0.000	0.000 (1, 355)	0.247	0.001	0.356 (1, 354)
						*p* =.994			*p* =.551

*B *unstandardized regression coefficient, *P* p-value, *PSC* psychosocial safety climate, *CI* confidence interval

Group: Control group as reference. Sex: male as reference. Occupational position: blue-collar workers as reference

## Discussion

This study aimed to investigate whether PSC acts as a moderator for the intervention effect of PT-A on (mental) health outcomes in a sample of employees in Germany. We examined two time points separately, i.e., nine and 15 months after enrolment in the study. It was observed, that at both time points, participants in the intervention group reported significantly better mental health in terms of a reduction in depressive symptoms and anxiety symptoms after the intervention, compared to the control group. No indications have been found that PSC might influence the magnitude of an intervention effect of PT-A on the outcomes studied (depressive symptoms, anxiety symptoms, general health status).

The results showed that participants in the intervention group reported significantly less depressive symptoms, anxiety symptoms and increased general health status, compared with the control group, at both time points. It is possible that these intervention effects may be underestimated as in our case, the control group received potentially more guidance and support than individuals in care as usual. The first diagnostic session with a psychotherapist and the following supportive phone call after three months may be regarded as professional help, which can motivate or help individuals to take action and explore possibilities that were mentioned to them. This may explain why not only symptoms were reduced in the intervention group but also in the control group. With randomization into the treatment groups and data collection at several time points it was tried to reduce a risk of regression to the mean. However, due to ethical reasons it was of importance to provide all study participants with a certain amount of support.

The present findings regarding the effect of PSC are novel and may be surprising, as a previous study showed that a high PSC contributed to better mental health and well-being after undergoing EAP [18]. The contrasting results may be due to the different nature of the interventions: While EAP is considered to be a multi-service offer including for example brief consultation with possible referral [20, 21], PT-A is specifically aimed at individuals suffering from symptoms of CMD with therapeutic sessions by psychotherapists. In addition, the authors showed that PSC was directly related to mental health and well-being [18]. In terms of direct effects, the present study could only show that higher PSC was associated with reduced anxiety symptoms at T1. For example, a previous study of the same study sample at baseline has found that PSC was negatively correlated with depressive and somatic symptoms, in a sub-sample of individuals working in large companies (i.e., companies with more than 250 employees) and it was suggested that PSC may be more relevant in larger companies as a flat hierarchy or shorter communication channels may be more relevant in smaller companies [5]. However, as the majority of participants were employed in larger companies we do not believe that consideration of only larger companies would alter our results. Although PSC is known to be a relevant factor in preventing stress and contributing to better psychological health [2], the non-significance of its influence on the intervention effect warrants further discussion.

A Swedish study has developed benchmarks against self-reported occupational safety and health (OSH) practices for the short version of the PSC, which are suggested to indicate risk levels of PSC among the Swedish working population [30]. A cross-culture validation of the PSC in Germany and Sweden confirmed the importance and usage of PSC in a European setting [32]. Although for example, differences in cognitive interviews were found regarding communication in relation to psychological health in the two countries [32], they both belong to the countries with the highest level in Europe of providing clear national guidelines for OSH [33]. Due to the shared directive 89/391 EEC of the EU, the two countries may be comparable when it comes to the importance of assessing and supporting a good PSC. Applying the benchmarks to our study samples, it can be seen that, on average, about 7% of participants at T1 and T2 reported a high risk, suggesting that there is an urgent need to improve risk management in relation to the working environment [30]. Approximately 85% of the individuals are in the moderate risk group. However, it must be taken into account, that these are selective samples of the respective companies, where a more negative perception of the PSC may be expected as the participants already experience health impairments. It seems that the individuals participating in the RCT report risk levels that would require their organizations to take action to promote PSC and OSH practices. Thus, although PSC may not play a major role in the level of success of PT-A in terms of (mental) health symptoms, it still needs attention in terms of promoting health and safety at work in general. For example, there are other areas, where previous research has found evidence that a high PSC helps indirectly to reduce psychological health problems [3, 7, 8]. These are for example reduction of bullying via implemented practices such as stress reduction or conflict resolution [3]. There are indications that certain workplace conflicts or problems (e.g. bullying), are better approachable when PSC is high [34]. One study found interaction effects for PSC and forms of stress-reporting stigma on bullying, indicating that in the context of high PSC, the positive association between stigma (supervisor and career-related) and experience of bullying was attenuated [34]. However, interactions effects have not been found for burnout [34]. The authors suggested that associations between stress-reporting stigma and burnout may be less easily influenced by PSC because of the individual-level nature of burnout which may contrast with the PSC as an organizational-level approach [34]. This may also be the case for our study findings on CMD symptoms or general health status: PSC, as an organizational level approach, may not lead to an impact on how individuals perceive their personal improvement or change in symptoms. This would underline the effectiveness of individual-oriented approaches, regardless of perceptions of the organizational structures. As stigmatization of CMD in the workplace is a major barrier to addressing mental health concerns and also to accessing treatment, it is important to focus on PSC and organizational culture in addition to individual level interventions [35]. It may be possible that a good PSC at work is beneficial in a way that employees are more open and start seeking help at work to cope with their demands or stressful situations, ideally before CMD may develop as consequences. For example, in high PSC-settings, employees may feel safer to utilize available resources [36]. Within a high PSC, it is suggested that the management should provide certain resources for the employees so that they feel supported to fulfill workplace demands [4]. In regard of this, research has shown that in a context of high PSC the association between demands and depression was diminished [37]. For example, more studies on a buffering effect of PSC showed that resources at work were stronger associated with reduction of psychological distress when PSC was high [38] or that negative effects of emotional demands on emotional exhaustion diminish under a context of high PSC [39]. It can therefore be concluded that the combination of psychosocial work factors and the organizational climate (PSC) may provide a good basis for reducing stress at work. In addition, interventions such as PT-A, which aim to improve coping with psychosocial work stressors on an individual, very personal basis, are needed and, as we have shown are effective in reducing depressive and anxiety symptoms.

### Limitations

The results should be considered with the following limitations in mind. As this study is a secondary analysis of a RCT, the results at hand need to be interpreted carefully due to the exploratory approach and lack of a-priori power analysis for the specific aim. However, the analyses entail important results which could be of interest for future interventional studies. As we have a very large sample size, the statistical power may be increased to detect a true effect and random error may be reduced. We could have detected effect sizes of f^2^ being 0.019 or greater, which indicates that there simply may be no effect for the association at hand or that the effect was too small to be detected. In addition, as the analyses were based on a pre-planned exploratory approach, we have not performed linear mixed effect models or multiple imputation. Participants were not asked during the RCT whether they had changed employers. If individuals changed employer after reporting their PSC at baseline, this might also go in hand with a change in PSC, which could bias the results. Similarly, individuals who are on sick leave for a long period may also not be able to accurately report the current PSC at their workplace. However, only a small number of individuals belong to this group (i.e., only *n* = 36 reported having more than 65 sick days at baseline). In addition, the study sample included individuals with managerial responsibilities and their reporting of a PSC may differ from that of individuals without managerial responsibilities. However, more than three quarters of the participants in the present study reported not having a managerial responsibility.

### Implications

The findings of the present study highlight the need for further research into PSC and its potential to support and protect the health and safety of employees. As there is a paucity of research investigating the construct of PSC in employees undergoing PT-A, further research onto its impact on employees’ mental health is warranted. In addition, as we have presented, the study samples showed moderate levels of PSC, in line with recommendations, suggesting the need for interventions to improve OSH practices in companies in Germany [30]. It is well known that a high PSC can contribute to better employee well-being [2], which suggests that companies should continue to strive to maintain a good working climate. However, as we have shown that PSC may not contribute to the magnitude of the effect of PT-A, there may be other factors that are more important and need to be investigated in further studies. These could be other psychosocial factors, such as the resources available at work or social support from colleagues or superiors. However, as we have shown an intervention effect on mental health symptoms with this secondary intervention approach, PSC may be a more helpful in terms of primary intervention approaches to help reducing the risks of development of CMD.

## Conclusions

The aim of the study was to investigate whether PSC moderates an intervention effect of PT-A on (mental) health outcomes. No interaction effects were found, suggesting that PSC does not play a role in the magnitude of symptom reduction when participating in PT-A. Nevertheless, offering PT-A to employees leads to a significant reduction in depressive and anxiety symptoms. There were also almost no main effects found for PSC on (mental) health outcomes. Future research needs to further explore whether PSC can support or protect employees who report to have poorer mental health.

## Supplementary Information

Supplementary Material 1.

Supplementary Material 2.

Supplementary Material 3.

## Data Availability

The datasets generated and/or analyzed during the current study are not publicly available due to data protection regulations, but are available from the study director on reasonable request.

## Abbreviations

CI: Confidence Interval
CMD: Common Mental Disorders
GAD-2: Generalized Anxiety Disorder Scale 2
EAP: Employee Assistance Program
PHQ-9: Patient Health Questionnaire 9
PSC: Psychosocial Safety Climate
PT-A: Psychotherapeutic consultation at work
RCT: Randomized Controlled Trial
VR-12: Veterans RAND 12-Item Health Survey
