# Supplementary material for: The impact of psychosocial safety climate on the intervention effect of psychotherapeutic consultation at work in Germany – secondary analysis of a randomized controlled trial
Source: BMC Public Health. 2025 Oct 22;25:3564. doi: 10.1186/s12889-025-24394-5 (PMC12541983; doi:10.1186/s12889-025-24394-5)
Supplement: Supplementary file 1 — Supplementary Material 1. [file 12889_2025_24394_MOESM1_ESM.docx]

Table S.1. Unadjusted regression analyses predicting depressive symptoms, anxiety symptoms and general health status at T1 and T2.

|  | **Depressive symptoms T1** | | |
| --- | --- | --- | --- |
|  | **Unadjusted model** | | |
|  | B | p | 95% CI |
| Depressive symptoms T0 | 2.713 | 0.00 | 2.25; 3.18 |
| Group | -2.44 | 0.00 | -3.37; -1.50 |
| PSC | 0.39 | 0.6 | -1.07; 1.86 |
| PSC*group | -0.54 | 0.26 | -1.49; 0.41 |
|  |  |  |  |
|  | Model fit | | |
|  | Adj. R^2^=0.274, F=39.714(4,406), p<.001 | | |
|  |  | | |
|  | **Depressive symptoms T2** | | |
|  | **Unadjusted Model** | | |
|  | B | p | 95% CI |
| Depressive symptoms T0 | 2.0 | 0.00 | 1.53; 2.46 |
| Group | -1.35 | 0.00 | -2.30; -0.40 |
| PSC | 0.78 | 0.29 | -0.68; 2.25 |
| PSC*group | -0.66 | 0.17 | -1.62; 0.29 |
|  |  |  |  |
|  | Model fit | | |
|  | Adj. R^2^=0.176, F=20.316(4,357), p<.001 | | |
|  |  | | |
|  | **Anxiety symptoms T1** | | |
|  | **Unadjusted model** | | |
|  | B | p | 95% CI |
| Anxiety symptoms T0 | 0.67 | 0.00 | 0.52; 0.82 |
| Group | -0.39 | 0.01 | -0.69; -0.09 |
| PSC | -0.14 | 0.56 | -0.62; 0.33 |
| PSC*group | -0.02 | 0.91 | -0.33; 0.29 |
|  |  |  |  |
|  | Model fit | | |
|  | Adj. R^2^=0.172, F=22.367(4,406), p<.001 | | |
|  |  |  |  |
|  | **Anxiety symptoms T2** | | |
|  | **Unadjusted model** | | |
|  | B | p | 95% CI |
| Anxiety symptoms T0 | 0.65 | 0.00 | 0.50; 0.80 |
| Group | -0.40 | 0.01 | -0.70; -0.09 |
| PSC | -0.15 | 0.53 | -0.62; 0.32 |
| PSC*group | 0.08 | 0.60 | -0.22; 0.39 |
|  |  |  |  |
|  | Model fit | | |
|  | Adj. R^2^=0.180, F=20.857(4,357), p<.001 | | |
|  |  | | |
|  | **General health status T1** | | |
|  | **Unadjusted model** | | |
|  | B | p | 95% CI |
| General health status T0 | 10.42 | 0.00 | 8.57; 12.26 |
| Group | 3.09 | 0.11 | -0.65; 0.84 |
| PSC | 1.60 | 0.60 | -4.28; 7.47 |
| PSC*group | -0.52 | 0.79 | -4.33; 3.28 |
|  |  |  |  |
|  | Model fit | | |
|  | Adj. R^2^=0.230, F=31.546(4,406), p<.001 | | |
|  |  | | |
|  | **General health status T2** | | |
|  | **Unadjusted model** | | |
|  | B | p | 95% CI |
| General health status T0 | 10.02 | 0.00 | 7.84; 12.17 |
| Group | 3.90 | 0.07 | -0.25; 8.03 |
| PSC | -0.88 | 0.79 | -7.27; 5.51 |
| PSC*group | 0.79 | 0.71 | -3.37; 4.95 |
|  |  |  |  |
|  | Model fit | | |
|  | Adj. R^2^=0.186, F=21.593(4,357), p<.001 | | |

B: unstandardized regression coefficient. P: p-value. PSC: psychosocial safety climate. CI: confidence interval.
