# Supplementary material for: The impact of psychosocial safety climate on the intervention effect of psychotherapeutic consultation at work in Germany – secondary analysis of a randomized controlled trial
Source: BMC Public Health. 2025 Oct 22;25:3564. doi: 10.1186/s12889-025-24394-5 (PMC12541983; doi:10.1186/s12889-025-24394-5)
Supplement: Supplementary file 2 — Supplementary Material 2. [file 12889_2025_24394_MOESM2_ESM.docx]

Table S.2. Pearson correlation between all study variables and time points (n=352-547).

|  | 1 | 2 | 3 | 4 | 5 | 6 | 7 | 8 | 9 | 10 | 11 | 12 | 13 | 14 |
| --- | --- | --- | --- | --- | --- | --- | --- | --- | --- | --- | --- | --- | --- | --- |
| 1 Age | 1 | -.105* | -.124** | .106* | -.076 | -.058 | .124* | .148** | -.036 | .078 | .081 | -.204** | -.272** | -.282** |
| 2 Sex |  | 1 | -.060 | .138** | -.028 | .058 | .042 | -.028 | .103* | .075 | .061 | .039 | -.057 | .027 |
| 3 Migration status |  |  | 1 | -.112** | .082 | 0.058 | .043 | .019 | .042 | -.003 | .011 | -.026 | -.050 | -.022 |
| 4 Occupational position |  |  |  | 1 | -.013 | -0.082 | -.050 | -.065 | .025 | .046 | .040 | .081 | .101* | .084 |
| 5 Psychosocial Safety Climate |  |  |  |  | 1 | -0.074 | -.115* | -.061 | -.050 | -.110* | -.042 | .055 | .066 | .032 |
| 6 Depressive symptoms T0 |  |  |  |  |  | 1 | .482** | .405** | .586** | .336** | .260** | -.414** | -.280** | -.244** |
| 7 Depressive symptoms T1 |  |  |  |  |  |  | 1 | .697** | .293** | .753** | .504** | -.331** | -.623** | -.440** |
| 8 Depressive symptoms T2 |  |  |  |  |  |  |  | 1 | .269** | .565** | .697** | -.272** | -.497** | -.582** |
| 9 Anxiety symptoms T0 |  |  |  |  |  |  |  |  | 1 | .397** | .419** | -.326** | -.178** | -.182** |
| 10 Anxiety symptoms T1 |  |  |  |  |  |  |  |  |  | 1 | .616** | -.252** | -.523** | -.361** |
| 11 Anxiety symptoms T2 |  |  |  |  |  |  |  |  |  |  | 1 | -.221** | -.402** | -.497** |
| 12 General health status T0 |  |  |  |  |  |  |  |  |  |  |  | 1 | .487** | .437** |
| 13 General health status T1 |  |  |  |  |  |  |  |  |  |  |  |  | 1 | .657** |
| 14 General health status T2 |  |  |  |  |  |  |  |  |  |  |  |  |  | 1 |
