# Supplementary material for: The impact of psychosocial safety climate on the intervention effect of psychotherapeutic consultation at work in Germany – secondary analysis of a randomized controlled trial
Source: BMC Public Health. 2025 Oct 22;25:3564. doi: 10.1186/s12889-025-24394-5 (PMC12541983; doi:10.1186/s12889-025-24394-5)
Supplement: Supplementary file 3 — Supplementary Material 3. [file 12889_2025_24394_MOESM3_ESM.docx]

Table S.3. Responder and non-responder differences in the sample.

| **T0 characteristics** | | **Responder** | | **Non-responder T1** | |  |
| --- | --- | --- | --- | --- | --- | --- |
|  |  | **Mean or n** | **SD** | **Mean or n** | **SD** | **p-value** |
| **Sex** | |  |  |  |  |  |
|  | Female | 235 |  | 61 |  | Chi-square test  0.629 |
|  | Male | 185 |  | 66 |  |  |
| **Age** | | 46.25 | 10.79 | 44.82 | 11.88 | t-test  0.203 |
| **Occupational position** | |  |  |  |  |  |
|  | Blue-collar workers | 88 |  | 37 |  | Chi-square test  0.053 |
|  | White-collar workers | 333 |  | 90 |  |  |
| **Psychosocial safety**  **climate** | | 1.58 | 0.93 | 1.57 | 1.00 | t-test  0.867 |
| **Depressive symptoms** | | 12.78 | 5.15 | 13.19 | 5.07 | t-test  0.429 |
| **Anxiety symptoms** | | 3.47 | 1.72 | 3.36 | 1.66 | t-test  0.534 |
| **General health status** | | 48.11 | 19.43 | 45.08 | 17.83 | t-test  0.118 |

Responder and non-responder were compared respectively for all available data (n=547). SD= standard deviation.

| **T0 characteristics** | | **Responder** | | **Non-responder T2** | |  |
| --- | --- | --- | --- | --- | --- | --- |
|  |  | **Mean or n** | **SD** | **Mean or n** | **SD** | **p-value** |
| **Sex** | |  |  |  |  |  |
|  | Female | 204 |  | 97 |  | Chi-square test  0.350 |
|  | Male | 167 |  | 79 |  |  |
| **Age** | | 46.56 | 10.68 | 44.58 | 11.71 | t-test  0.058 |
| **Occupational position** | |  |  |  |  |  |
|  | Blue-collar workers | 75 |  | 50 |  | Chi-square test  0.036 |
|  | White-collar workers | 296 |  | 127 |  |  |
| **Psychosocial safety**  **climate** | | 1.60 | 0.94 | 1.55 | 0.97 | t-test  0.576 |
| **Depressive symptoms** | | 12.60 | 5.25 | 13.45 | 4.84 | t-test  0.070 |
| **Anxiety symptoms** | | 3.45 | 1.75 | 3.44 | 1.61 | t-test  0.484 |
| **General health status** | | 47.97 | 18.46 | 46.21 | 20.37 | t-test  0.316 |

Responder and non-responder were compared respectively for all available data (n=547). SD= standard deviation.
